# An Automated System for Skeletal Maturity Assessment by Extreme Learning Machines

**DOI:** 10.1371/journal.pone.0138493

**Published:** 2015-09-24

**Authors:** Marjan Mansourvar, Shahaboddin Shamshirband, Ram Gopal Raj, Roshan Gunalan, Iman Mazinani

**Affiliations:** 1 Faculty of Computer Science and Information Technology, University of Malaya, Kuala Lumpur, 50603, Malaysia; 2 Faculty of Medicine, University of Malaya, Kuala Lumpur, 50603, Malaysia; 3 Faculty of Engineering, University of Malaya, Kuala Lumpur, 50603, Malaysia; Jiangnan University, CHINA

## Abstract

Assessing skeletal age is a subjective and tedious examination process. Hence, automated assessment methods have been developed to replace manual evaluation in medical applications. In this study, a new fully automated method based on content-based image retrieval and using extreme learning machines (ELM) is designed and adapted to assess skeletal maturity. The main novelty of this approach is it overcomes the segmentation problem as suffered by existing systems. The estimation results of ELM models are compared with those of genetic programming (GP) and artificial neural networks (ANNs) models. The experimental results signify improvement in assessment accuracy over GP and ANN, while generalization capability is possible with the ELM approach. Moreover, the results are indicated that the ELM model developed can be used confidently in further work on formulating novel models of skeletal age assessment strategies. According to the experimental results, the new presented method has the capacity to learn many hundreds of times faster than traditional learning methods and it has sufficient overall performance in many aspects. It has conclusively been found that applying ELM is particularly promising as an alternative method for evaluating skeletal age.

## Introduction

Skeletal maturity assessment, or bone age assessment (BAA), is a radiological process to examine the ossification development in the left-hand wrist and estimate the bone’s age by making comparisons with an atlas comprising hundreds of standard images [1]. Many disease in children such as growth disorders, chromosomal disorders, endocrine disorders and endocrinological problems could be discovered by the discrepancy between the bone age and chronological age. Bone age assessment is an important process in clinical routine; however, it has not improved much over the last 35 years [2,3]. There are two well-known methods applied for BAA: the Greulich-Pyle (GP) [4] and Tanner-Whitehouse (TW2) methods [5]. In the GP system, radiologists compare hand bone radiographs with standardized radiographs from the atlas and make evaluations, while the TW2 system is based on a scoring method [6]. The results from both these assessment types are associated with human observation variability, since a radiologist doing a bone age assessment to evaluate a child’s maturation cannot be certain about estimation accuracy [7,8]. Therefore, this has been the greatest motivator for presenting an automated method of estimating skeletal maturity (bone age)[9]. However, the computerized BAA system is still under the empirical period because of the inadequate performance of the system [10]. Some proposed methods have been discussed in the literature.

### State of art

The first try to design an automated system for bone age assessment have been reported by Nelson and Micheal, in 1989 [11]. Their system converted the images to the binary format and normalized the image before processing. This system have been never evaluated in a large scale due to the some drawback in overlapping of pixel intensity of bone in image processing technique. Manos with his team posed segmentation and presented a method for merging region and edge detection in pre-processing level [12]. However, the output of edge detection was not reliable and threshold was included the results. Pietka et al. [13] have designed a method based on analysis of carpal bone in hand-wrist. The system used dilation method to extract the carpal. Their research team improved the system by windowing technique to calculate the statistical features. However the new version of system still doesn’t find the solution for the segmentation problem. Another system was reported by Mahmoodi, has applied the binary thresholding and location searching using concave-convex followed by segmentation based on the active shape technique [14]. Sebastian et al. [15] has conducted a study on image segmentation base on deformable method, the pre-processing contained the region growing and local competition in region sections. The output of this system was acceptable but it was included the heavy computing processes and complicated calculating. In the system presented by Gertych et al. [16] adaptive segmentation technique was applied based on Gibbs random in the pre-processing stage. Zhang et al. [17] worked on the carpal segmentation using the anisotropic diffusion and adaptive image threshold in the pre-processing stage. The proposed included canny edge detection that is not robust technique in image segmentation. Han et al. [18] presented Gradient vector flow (GVF) to use the segmentation while this technique was involved heavy loading process for edge detection. Liue et al. [19] suggested primitive image processing method that is similar to edge detection and simulate matching at the pre-processing level of segmentation. Most of the method is presented to the model for segmentation of the hand, however estimation of bone age according this method was never assess accurately. Hence, this method cannot be introduced as a fully automated system for bone age assessment.

Bone age analysis requires high accuracy for assessment. The aim of this study is to introduce a new model of determining bone age based on content-based image retrieval (CBIR) technique as a part of a novel age assessment method, using a soft computing approach, namely extreme learning machines (ELM) for evaluation, which is a 100% automated method for BAA. Nowadays, applying modern computational approaches to solving real problems and determining optimal values and functions has been receiving enormous attention from researchers in diverse scientific disciplines [20]. Neural networks (NN), a vital computational approach, has recently been introduced and applied in various engineering areas such as medical application diagnosis [21,22]. This method facilitates solving complex nonlinear problems, which are otherwise difficult to solve with classic parametric methods. There are numerous algorithms for training neural networks, such as hidden Markov model (HMM), back propagation, and the support vector machines (SVM). A shortcoming of NN is learning time application. Huang et al. [23] introduced an approach for single-layer feed forward NN known as Extreme Learning Machines (ELM). This technique is capable to solve the problems are creating by gradient descent-based algorithms like backpropagation in ANNs. ELM can decrease the required time for training a Neural Network. In fact, it has been proven that by using ELM, the learning process becomes very fast and generates robust performance [24]. Accordingly, a number of investigations have been carried out related to the successful application of the ELM algorithm in solving problems in various scientific fields [25–30].

In general, ELM is a powerful algorithm with faster learning speed than traditional algorithms like backpropagation (BP) and superior performance. ELM attempts to achieve the standard of weights with the smallest error rate of training.

In this study, a new automated bone age assessment approach is developed and evaluated by the ELM measurement and elimination the need for image segmentation. The results indicate that the proposed model can adequately estimate skeletal age. The ELM results are also compared with the results from genetic programing (GP) and artificial neural networks (ANNs). An attempt is made to retrieve the correlation between chronological age and bone age.

## Methodology

**Fig 1:**
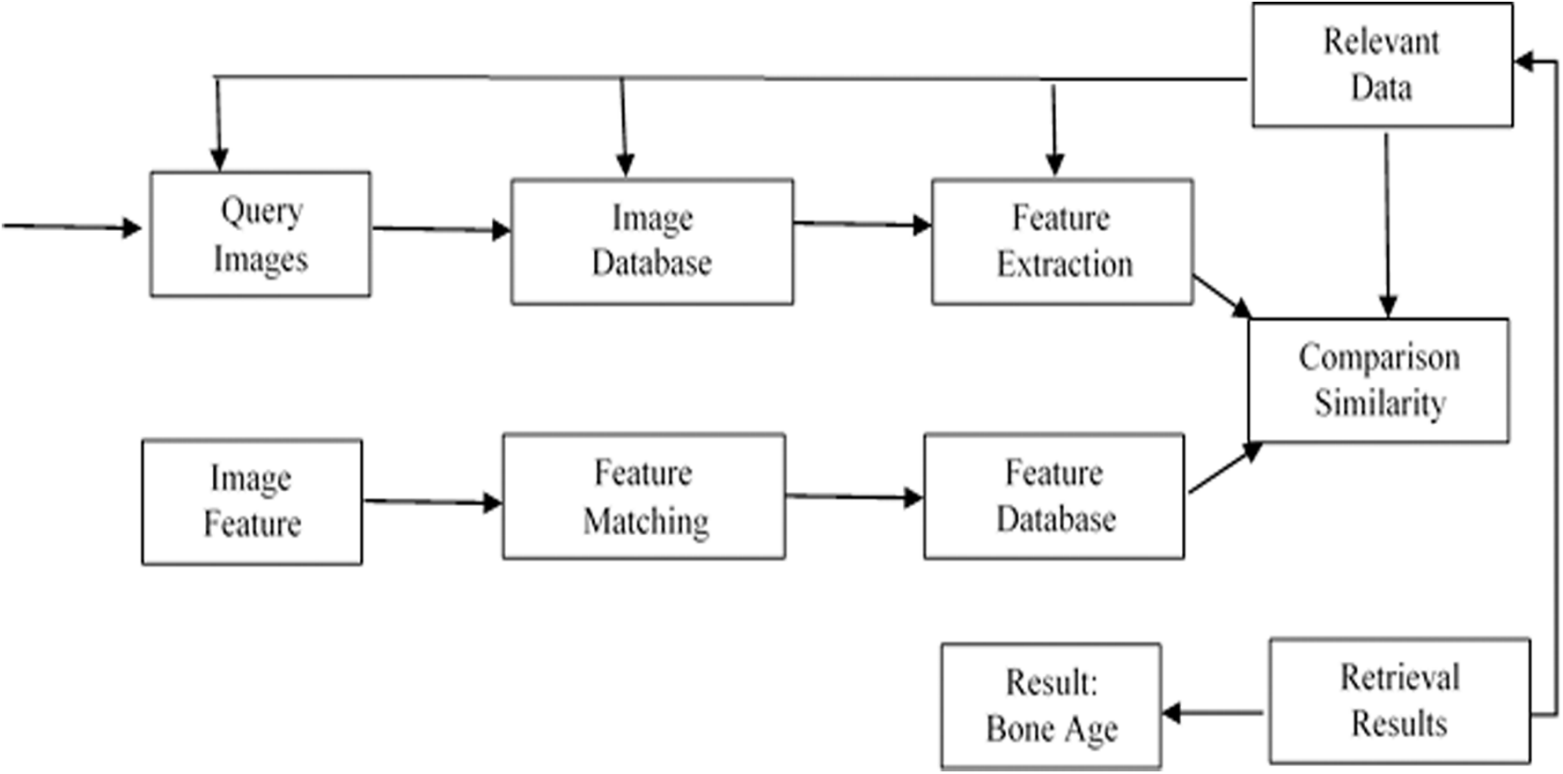
CBIR layout in the bone age assessment system.

The Content-based image retrieval (CBIR) approach is become famous in medical imaging as well as crime prevention in recent years [31]. The CBIR system was developed in the 1990s to solve problems encountered in text-based image retrieval. The CBIR method is based on querying by image [32]. Content-based image retrieval is a robust method to determine age independent of bone measurements. The CBIR methodology for skeletal age assessment is involves comparing image content for a new input with earlier samples. Most BAA systems are applied to the regions of interest (ROIs) in hand bones, which leads to low accuracy in bone age assessment [17,33,34]. The new method utilized in our study overcomes the mentioned limitation in literature by using complete images for an individual query instead of applying the query to the regions of interest (ROIs) [35]. The CBIR assessment methodology is found on compressing image content from a new sample with the earlier samples. Fig 1 shows the CBIR layout applied in our BAA system.

In our system, not only are whole images considered, but so is visual content information such as ethnicity and gender, since these features allow the system to correctly perform extractions from the available data. The feature extraction includes getting the related features from images. Therefore, the features are extracted from the hand radiographs, and an optimal subset of the selected features is picked. Feature extraction is based on the Weighted PCA as it is one of the best pattern recognition methods in computer vision applications [36]. It is certainly the most suitable feature extraction method for this study as it is a linear feature extraction technique [37] that is both efficient and fast. The retrieval images in this research included third party data provided from the database in Medical Image Research Centre (IRMA) available at “https://ganymed.imib.rwth-aachen.de/irma/institute_irmadaten.php”. There are 1100 X-rays classified as female and male, and four ethnicities: Asian, Caucasian, African/American and Hispanic [38].

### Age assessment

**Fig 2:**
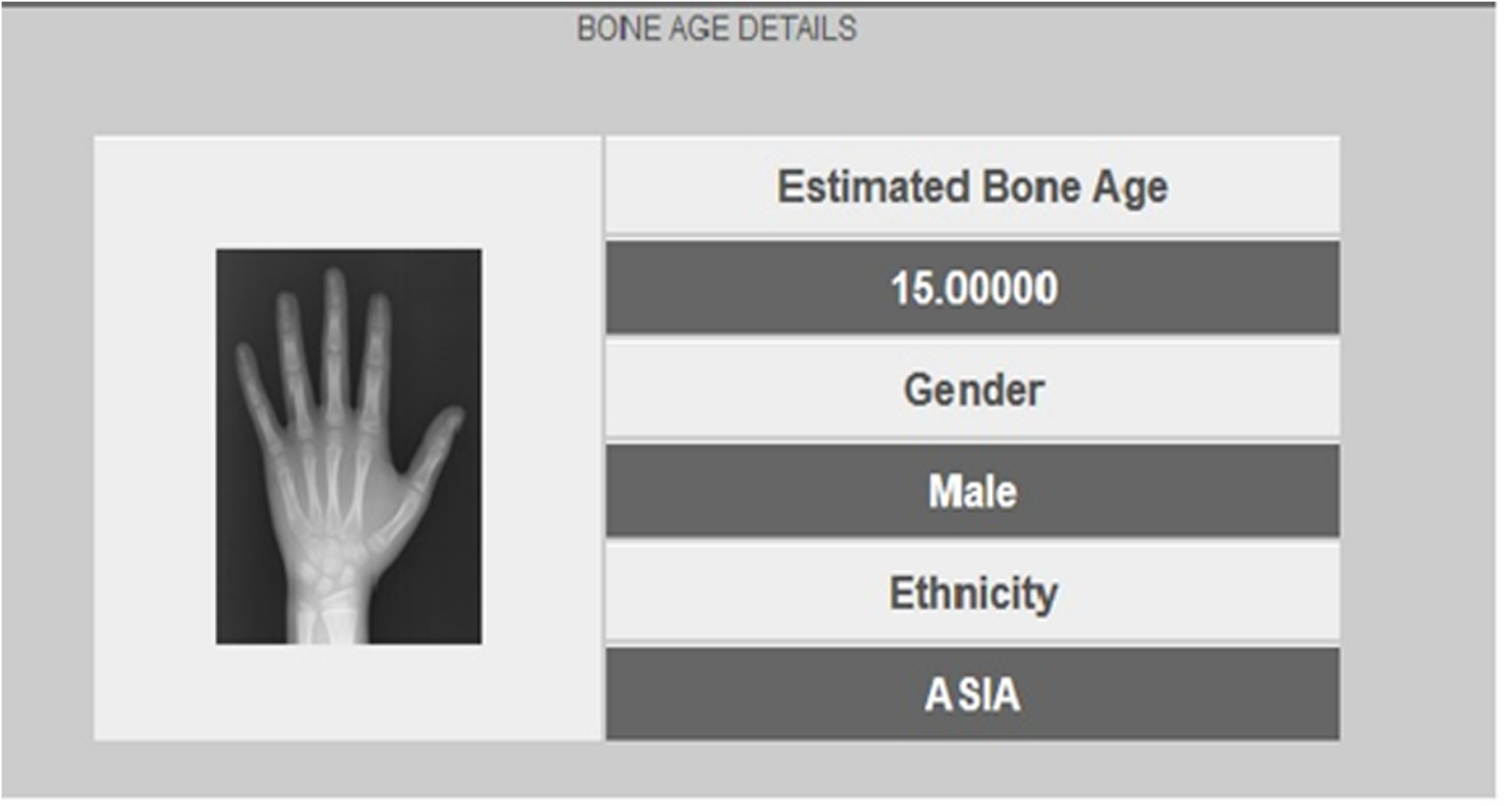
Bone age result displayed with the gender and ethnicity features.

The main step in implementing our BAA system is the process of estimating bone age according to the automated technique (Fig 2). Bone age is assessed by comparing a radiograph with samples from a repository that contains various ages for both genders and four different ethnicities. A temporary repository is needed to rank the retrieved radiographs. The tagged age values of the retrieved images are utilized as part of the BAA process and the final estimated age is calculated as the mean of the retrieved values:

Predicted Bone Age = $\frac{\sum_{i=1}^{n}x}{n}$

*Where x* = Age of highest ranked retrieved images

*n* = Total number of highest ranked retrieved images

Therefore, bone age assessment is computed in the following steps:

1. Related features are extracted and stored in the database for a temporary period.
2. An individual query is enforced to the system’s search engine by each feature.
3. The best matching output is retrieved from the feature repository according the similarity score for the query.

### Validation Experiments

The image data used for the evaluation consists of images collected from normal samples. The age range of the images is 1–18 years for both genders, male and female. The radiographs are classified and scanned in X-ray format with 256 x 260 pixel size. Tables 1 and 2 illustrate the input variables and output results used to validate our system in terms of definition and obtained values.

**Table 1:** Category numbers of samples used for evaluation.

Age group	Category							
	AF	AM	CF	CM	AAF	AAM	HF	HM
1–6	4	4	4	4	4	3	4	4
7–12	3	4	3	4	3	4	3	4
13–18	4	4	4	3	4	4	4	4

Ethnicity is denoted by

*A*: Asian

*C*: Caucasian

*AA*: African American

*H*: Hispanic; and Gender is

F: Female

*M*: Male

**Table 2:** Evaluation of the BAA system based on the comparison with chronological age.

	Asian		Caucasian		African/American		Hispanic	
	Female	Male	Female	Male	Female	Male	Female	Male
System bone age (Mean)	9.036	10.615	11.036	9.2700	8.244	11.881	10.414	8.696
Chronological age (Mean)	9.102	10.983	10.695	9.390	8.188	12.023	10.266	8.003
Number of cases	11	12	11	11	11	11	11	12

### Extreme Learning Machines (ELM)

Huang at el. [23] introduced the extreme learning machine (also called ELM) according the single-layer feed-forward neural network (SLFN) structure as a tool for learning algorithms [39,40]. The ELM solved the problems like improper learning rate, local minima, and over fitting commonly in iterative learning approaches [41]. ELM selects the input weights randomly and decides the output weights of SLFN analytically. ELM includes a more favourable general capability with faster learning speed. This algorithm does not require much human intervention and can execute much faster than other customary algorithms. The ELM algorithm is able to analytically specify all network variables that prevent human intervention. ELM is an effective technique with numerous advantages including high performance, ease of use, rapid learning speed, kernel functions and suitability for nonlinear activation.

#### Single hidden layer feed-forward neural network (SLFN)

SLFN structures include *L* hidden nodes which are usually applied like a mathematical theory of SLFN, combination of the two additives and RBF hidden nodes in an integrated way [42,43]:

$$f_{L}\left(x\right)=\sum_{i=1}^{L}\beta_{i}G\left(a_{i},b_{i},x\right),\;x\in R^{n},\;a_{i}\in R^{n}$$ (1)

In the Eq (1) the learning variables of the hidden nodes indicated by *a*_*i*_ and *b*_*i*_ respectively; the weight joining that presented by *β*_*i*_ *is* the *i*th hidden node toward the output node; and the output value of the *i*th hidden node in related to the input *x* is *G*(*a*_*i*_, *b*_*i*_, *x*). The additive structure *g*(*x*): *R* → *R* (e.g., sigmoid and threshold), *G*(*a*_*i*_, *b*_*i*_, *x*) by the activation, hidden node is:

$$G\left(a_{i},b_{i},x\right)=g\left(a_{i}.x+b_{i}\right),\;b_{i}\in R$$ (2)

where *a*_*i*_ represents the vector of the weight that connects the input layout to the *i*th hidden node; *b*_*i*_ is the basis of the *i*th hidden node *a*_*i*_; *x* is the inner vector result *a*_*i*_ and *x* in *R* ^*n*^. *G*(*a*_*i*_, *b*_*i*_, *x*) can be found for the RBF hidden node with the activation structure *g*(*x*): *R* → *R* (e.g., Gaussian), *G*(*a*_*i*_, *b*_*i*_, *x*) as [39]:

$$G\left(a_{i},b_{i},x\right)=g\left(b_{i}\left\|x-a_{i}\right\|\right),\;b_{i}\in R^{+}$$ (3)

Since *a*_*i*_ and *b*_*i*_ demonstrate the centre as well as the impact factor of the *i*th RBF node. The series of all positive real parameters presented by *R* ^*+*^. In addition, the RBF network considers as a particular case of SLFN with RBF nodes in its hidden layer. For *N* arbitrary distinct samples (*x*_*i*_, *t*_*i*_) ∈ *R* ^*n*^ × *R* ^*m*^, *x*_*i*_ is the *n* × 1 input vector and *t*_*i*_ is the *m* × 1 target vector. While an SLFN with *L* hidden nodes could be predict these *N* samples with zero error, it suggests there exist *β*_*i*_, *a*_*i*_ and *b*_*i*_ like as [39]:

$$f_{L}\left(x_{j}\right)=\sum_{i=1}^{L}\beta_{i}G\left(a_{i},b_{i},x_{j}\right),\;j=1,\ldots .,N.$$ (4)

Eq (4) could be computed compactly as:

Hβ=T (5)

Where

$$H(\tilde{a},\tilde{b},\tilde{x})={\left[\begin{array}{ccc} G\left(a_{1},b_{1},x_{1}\right) & \cdots & G\left(a_{L},b_{L},x_{1}\right)\\ \; & \cdots & \;\\ \left(a_{1},b_{1},x_{N}\right) & \cdots & G\left(a_{L},b_{L},x_{N}\right) \end{array}\right]}_{N\times L}$$ (6)

with $\tilde{a}=a_{1},\ldots ,a_{L};\;\tilde{b}=b_{1},\ldots ,b_{L};\;\tilde{x}=x_{1},\ldots ,x_{L}$

$$\beta ={\left[\begin{array}{c} \beta_{1}^{T}\\ \vdots \\ \beta_{L}^{T} \end{array}\right]}_{L\times m}\;\text{and}\;T={\left[\begin{array}{c} t_{1}^{T}\\ \vdots \\ t_{L}^{T} \end{array}\right]}_{N\times m}$$ (7)

Where *H* is the hidden level of result matrix of SLFN with the *i*th column of *H* being the *i*th hidden node’s output related to inputs *x*_1_,…, *x*_*N*_.

#### Principles of ELM

Recently, the application of ELM have been extensively studied in different research domains especially in biomedical engineering. ELM has three bold features from learning efficiently point of view: high learning accuracy, fast learning speed and least human invention. The benefit of ELM in generalization over traditional algorithms has been proved for the problems from various areas [44]. The algorithms introduced in neural networks do not included the generalization efficiency when they are applied for the first time. While, ELM reached the better generalization efficiency by the smallest training error rate. It was for this reason that we used ELM as it had the best chance to provide us with improved results.

ELM was defined as a SLFN by *L* hidden neurons is able to learn *L* distinct samples which has zero error [27]. Even with the number of hidden neurons (*L*) is less than the number of distinct cases (*N*), ELM can still assign random parameters to the hidden nodes and compute the output weights by the pseudo inverse of *H*, with only a small error of ε > 0. The hidden node variables of ELM *a*_*i*_ and *b*_*i*_ can easily be adjusted random parameters and also they should not be tuned throughout training. These notions will be defined in the following theorems:

*Theorem 1*: Let there be an SLFN with *L* additive or RBF hidden nodes and an activation structure *g*(*x*) that is extremely differentiable in all interval of *R*. Furthermore, for arbitrary *L* definite input variables {*x*_*i*_ | *x*_*i*_ ∈ *R* ^*n*^, *i* = 1,…, *L*} and ${\left\{\left(a_{i},b_{i}\right)\right\}}_{i=1}^{L}$ randomly created by all continuous possibility distribution, respectively, the hidden layer output matrix is invertible with the probabilities of one, and the hidden layer output matrix *H* of the SLFN is invertible and ‖*Hβ*−*T*‖ = 0.

*Theorem 2*: (Liang et al. [34]) Assigning the small positive rate of ε > 0 and activation operation *g*(*x*): *R* → *R*, that is considerably differentiable in any interval, presently there is existent *L* ≤ *N* like that for *N* arbitrary distinct input vectors {*x*_*i*_ | *x*_*i*_ ∈ *R* ^*n*^, *i* = 1,…, *L*} for each ${\left\{\left(a_{i},b_{i}\right)\right\}}_{i=1}^{L}$ randomly produced based upon any continuing potential distribution ‖*H*_*N*×*L*_ *β*_*L*×*m*_ − *T*_*N*×*m*_‖ < *ε* with a probability of one.

As the hidden node variables of ELM cannot be adjusted throughout training since, they are allocated with random parameters, Eq (5) becomes a linear algorithm and the output weights should be appraised like the following [39]:

$$\beta =H^{+}T$$ (8)

Since *H* ^+^ indicated the Moore-Penrose generalized inverse [45] of the hidden level output matrix H which could be computed with many approaches containing orthogonal projection, orthogonalization, iteration, singular value decomposition (SVD), etc. [45]. The orthogonal projection technique is utilized only when *H* ^*T*^ *T* is non-singular and H^+^ = (*H* ^*T*^ *T*)^−1^ *H* ^*T*^. Owing to the presence of searching and iterations, orthogonalization and iteration methods are included limitations. Implementations of ELM are based on SVD to compute the Moore-Penrose generalized inverse of *H*, because it can be used in all positions. Hence, ELM is considered a batch learning method.

### Artificial neural networks

The backpropagation learning algorithm in the multilayer feedforward network presented the famous neural network structures [46], it is widely used in different scientific fields [47]. Ordinarily, a neural system contains of three levels: (i) an input level; (ii) a middle or hidden level; and (iii) an output level. The first directions are *D* = (*X*_1_, *X*_2_, …, *X*_*n*_)^*T*^ and *D* ∈ *R* ^*n*^; the outputs of *q* neurons in the hidden level are presented by *Z* = (*Z*_1_, *Z*_2_, …, *Z*_*n*_)^*T*^; and finally the results of the output level include the *Y* ∈ *R* ^*m*^, *Y* = (*Y*_1_, *Y*_2_, …, *Y*_*n*_)^*T*^. Adopting the tolerance among the input and hidden levels and also the weight are shown by *w*_*ij*_ and *y*_*j*_ respectively and the weight and assuming thorough the hidden and output layers are presented by *w*_*jk*_ and *y*_*k*_ respectively, furthermore the outputs of any neuron in a hidden level and also output level are represented as following:

$$Z_{j}=f\left(\sum_{i=1}^{n}w_{ij}X_{i}-\theta_{j}\right)$$ (9)

$$Y_{k}=f\left(\sum_{j=1}^{q}w_{kj}Z_{j}-\theta_{k}\right)$$ (10)

Since *f* is applied as transfer function, is included the rule for planning the neuron’s summed input to its output, using a proper instrument for providing non-linearity to the network system. The sigmoid function represents a major function that is monotonic improving and changing from zero to one.

### Genetic programming

Genetic programming, or GP, is a progressive method containing Darwinian principles about natural parameters and survival to predict statement in symbol style. GP programming defines how the outputs relate to the input variables. This technique utilizes a basic sample of randomly generated programs (equations) extracted from a random mix of input values, functions and random numbers including arithmetic operators, comparison/logical functions and mathematical functions, which must be selected according to understanding of the process properly. Some solutions are exposed to the evolutionary procedure and the ‘fitness’ of the developed programs is examined. Particular programs with the best data fit are then picked up from the basic population sample. The structures that are the best matches choose to change some of the data among themselves to create better structures from ‘mutation’ and ‘crossover,’ which imitate the reproduction process in the natural world. In the genetic algorithm, mutation means exchanging programs randomly to make new structures, and crossover refers to the changing sections of the best programs with each other. This development routine repeat over successive generations and drive towards searching data for symbolic expressions that could be scientifically clarified to derive procedure information. GP provided a big improvement in the computer science, chemistry, bioinformatics, engineering and mathematics by the metaheuristic (called search heuristic) technique [48–50].

## Results

### Proposed model accuracy evaluation

The performance of the proposed models is represented as root mean square error (RMSE), coefficient of determination (R^2^) and the Pearson coefficient (r). These statistics are defined as follows:

root-mean-square error (RMSE)

$$RMSE=\sqrt{\frac{\sum_{i=1}^{n}{\left(P_{i}-O_{i}\right)}^{2}}{n}}\;,$$ (11)

Pearson correlation coefficient (r)

$$\text{r}=\frac{\text{n}\left(\sum_{\text{i}=1}^{\text{n}}{\text{O}}_{\text{i}}\cdot {\text{P}}_{\text{i}}\right)-\left(\sum_{\text{i}=1}^{\text{n}}{\text{O}}_{\text{i}}\right)\cdot \left(\sum_{\text{i}=1}^{\text{n}}{\text{P}}_{\text{i}}\right)}{\sqrt{\left(\text{n}\sum_{\text{i}=1}^{\text{n}}{\text{O}}_{\text{i}}^{2}-{\left(\sum_{\text{i}=1}^{\text{n}}{\text{O}}_{\text{i}}\right)}^{2}\right)\cdot \left(\text{n}\sum_{\text{i}=1}^{\text{n}}{\text{P}}_{\text{i}}{}^{2}-{\left(\sum_{\text{i}=1}^{\text{n}}{\text{P}}_{\text{i}}\right)}^{2}\right)}}$$ (12)

coefficient of determination (R^2^)

$${\text{R}}^{2}=\frac{{\left[\sum_{\text{i}=1}^{\text{n}}\left({\text{O}}_{\text{i}}-\bar{{\text{O}}_{\text{i}}}\right)\cdot \left({\text{P}}_{\text{i}}-\bar{{\text{P}}_{\text{i}}}\right)\right]}^{2}}{\sum_{\text{i}=1}^{\text{n}}\left({\text{O}}_{\text{i}}-\bar{{\text{O}}_{\text{i}}}\right)\cdot \sum_{\text{i}=1}^{\text{n}}\left({\text{P}}_{\text{i}}-\bar{{\text{P}}_{\text{i}}}\right)}$$ (13)

While *O*_*i*_ and also *P*_*i*_ are the assessed value of bone age and the experiential, accordingly, further more *n* refers to the total amount of tested data.

### Performance evaluation of the proposed ELM model

**Fig 3:**
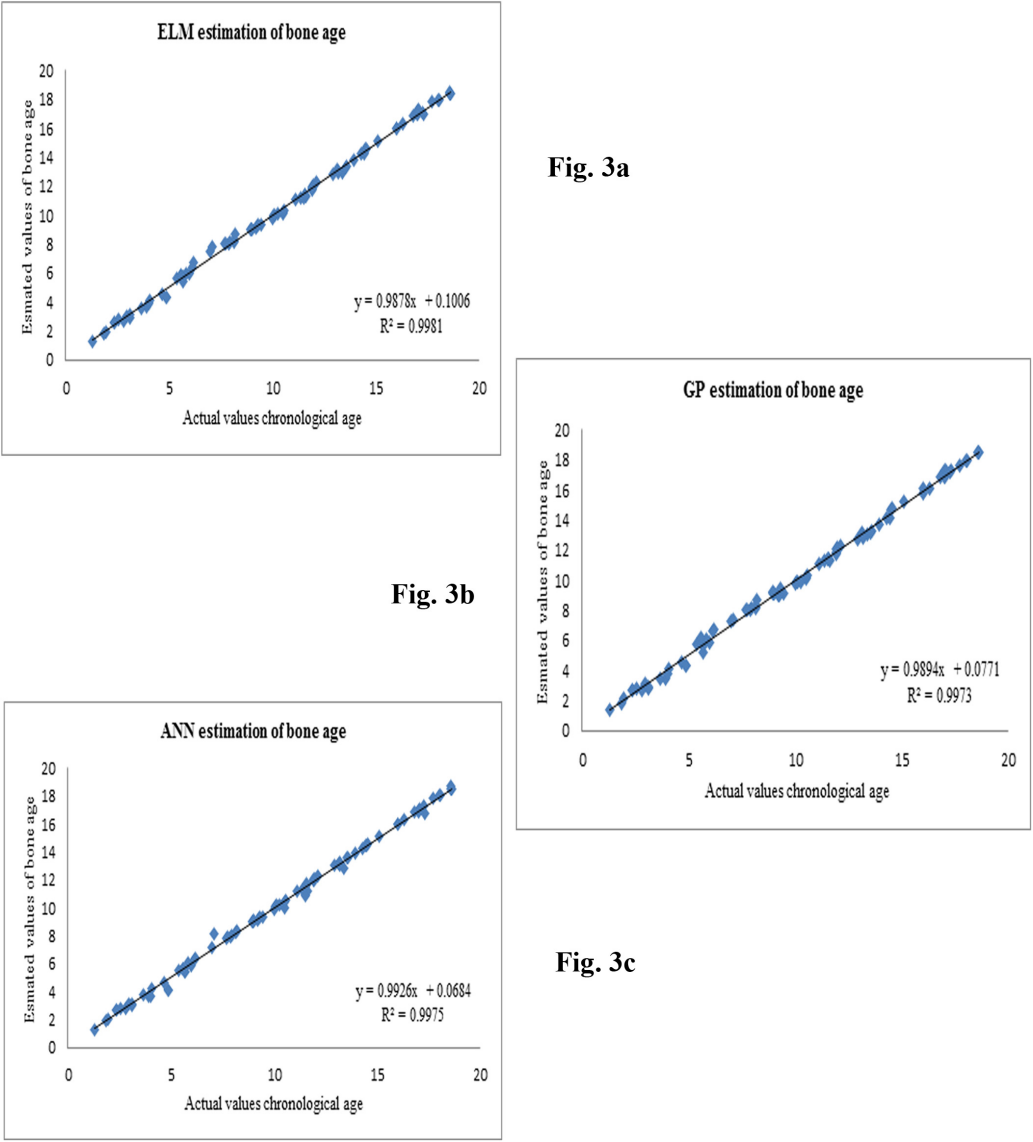
Scatter plots of actual and estimated bone age values using (a) ELM, (b) GP and (c) ANN.

**Fig 4:**
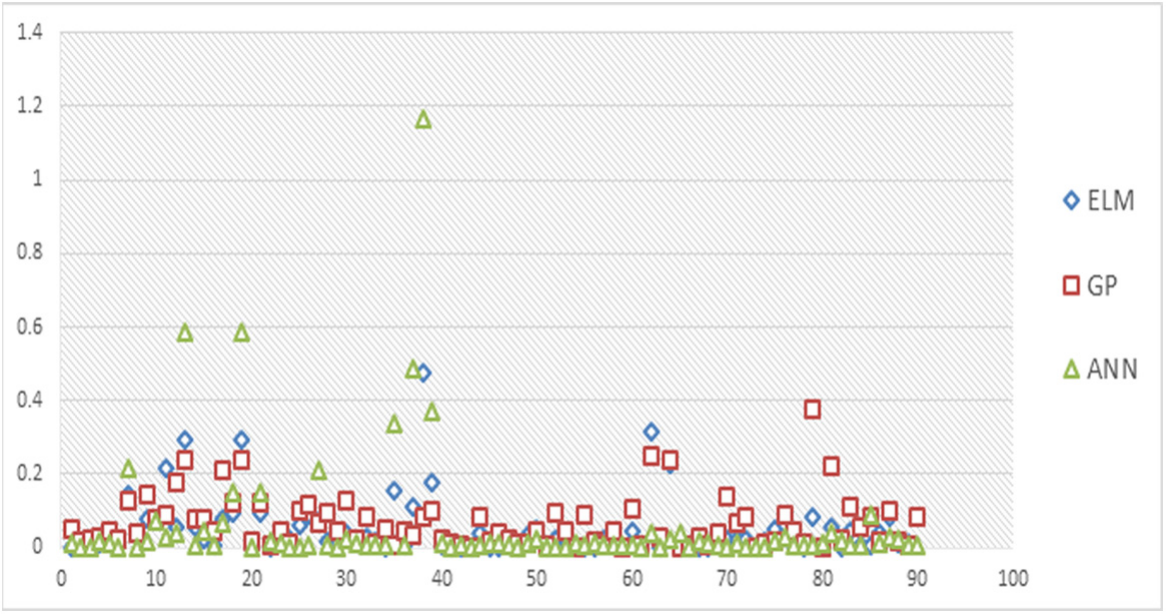
Comparison of error rate for the soft computing models.

This section reports the results of the ELM bone age assessment models. Fig 3A shows the accuracy of the presented ELM BAA model. Subsequently, Fig 3B and 3C present the accuracy of the GP and ANN BAA models, respectively. It can be seen that most of the points fall along the diagonal line for the ELM assessment model. Consequently, the estimation results are in very good agreement with the measured values for the ELM model. This observation is supported by the very high coefficient of determination value. The number of either overestimated or underestimated values produced is limited. Thus, it is obvious that the estimated values exhibit high precision levels. Fig 4 shows the comparisons of error rates for the three soft computing models used in this study.

### Architecture of soft computing models

The parameters of the ELM, ANN and GP modelling frameworks employed in this study are presented in Table 3.

**Table 3:** User-defined parameters for the ELM, ANN and GP models.

ELM		ANN		GP	
Number of layers	3	Number of layers	3		
Neurons	Input: 3; Hidden: 3, 6, 10; Output: 1	Neurons	Input: 3; Hidden: 3, 6, 10; Output: 1	Neurons	Output: 1
		Number of iteration	1000	Population size	512
		Activation function	Sigmoid Function	Function set	+,-,×,÷,√, ln, $\overset{x}{e},\;\overset{x}{a}$
Learning rule	ELM for SLFNs	Learning rule	Back propagation	Head size	5–9
				Chromosomes	20–30
				Number of genes	2–3
				Mutation rate	91.46
				Crossover rate	30.56
				Inversion rate	108.53

## Discussion

### Performance comparison of ELM, ANN and GP

To demonstrate the merits of the presented ELM approach on a more definite and tangible basis, the accuracy of ELM model estimation was compared with the accuracy of estimation of the GP and ANN methods, which served as a benchmark. Conventional error statistical indicators, i.e., RMSE, r and R^2^, were used for comparison. Table 4. summarizes the results of estimation accuracy for the test datasets, since training error is not a credible indicator of the prediction potential of a particular model.

**Table 4:** Comparison of performance statistics of the ELM, ANN and GP bone age assessment models.

ELM			ANN			GP		
RMSE	R^2^	r	RMSE	R^2^	r	RMSE	R^2^	r
0.221247	0.9981	0.999025	0.241835	0.9975	0.998773	0.255281	0.9973	0.998669

The ELM model outperformed the GP and ANN models according to the results in Table 4. The ELM model provided significantly better results than the benchmark models. According to RMSE analysis in comparison with ANN and GP, it may be concluded that the proposed ELM outperformed the benchmark models. As ELM is a data driven algorithm, the primary limitation of our method is that it is heavily reliant on the data selection process.

## Conclusion

In this study, a systematic approach was carried out to create a new fully automated method to assess bone age using an ELM model, in depended to image segmentation. The ELM measurement was compared with GP and ANN in order to evaluate the models’ accuracy. The results calculated in terms of RMSE, r and R^2^, indicate that the ELM approach is superior to GP and ANN. Furthermore, the results revealed the robustness of the method.

The proposed system has many appealing, remarkable features that distinguish it from conventional, well-known gradient-based learning approaches for feedforward neural networks. ELM approach has much faster learning speed compared to traditional feedforward network learning algorithms such as backpropagation (BP). Moreover, unlike traditional learning algorithms, ELM is able to attain the standard of weights as well as the smallest training error. Future work will involve further improving the skeletal age assessment accuracy by expanding the database of images.
